# Indents

**Published:** 1916-01

**Authors:** 


### INDENTS

**☞ Brief Articles of Technical or Scientific Value will receive notice and comment. Contributions invited.**

*Cleaning Glassware.* Stained slides and glassware are readily cleaned by boiling for one minute in a mixture of equal parts of a six per cent. solution of sodium hydroxid and a two per cent. solution of calcium carbonate. The articles are then rinsed in cold water and wiped.—A. H. Kaplan, *Journ. Amer. Med. Association.*

*Encroaching on Live Dentine.* Until we can devise a bridge attachment by which the contact of the mechanical construction with the living tissue is brought down to the degree of perfection demanded by the man who puts in a contour gold filling so shaped that it shall not be productive of irritation, we have to contend with a constant pathological menace.—Dr. Kirk in *Cosmos*, Dec., 1915, page 1349.

*Notes on the Insertion of Silicate Cement Fillings.* Time can be saved while a silicate cement filling is setting, if the rubber dam be folded over the incisal edges of the teeth and tied with floss silk. By this means the filling can be kept dry, and other work in the mouth can be proceeded with.

When inserting a silicate cement filling in an approximal cavity, where the space is too narrow for the employment of an agate or bone spatula, a perfectly efficient instrument can be readily prepared in a few moments by whittling a stick of hickory to the desired thickness. This wood is dense and supple, and it will be found to work perfectly for the purpose required.—*Edwards' Dental Quarterly.*

*Injudicious Use of Hydrogen Dioxid.* I have seen so many cases of severe infections following the use of hydrogen dioxid, and still find so many men employing it, that I wish to impress upon those present the necessity of aiding in the reform of those who still persist in using it. Within the past four years I have seen it employed in treating apical and other abscesses; introduced into pyorrheal pockets; injected into suppurating sockets from which abscessed teeth have been extracted. The results of such treatment are extensive local inflammation too frequently followed by necrosis and osteomyelitis, and occasionally a general systemic infection which is often fatal. The use of this preparation in any suppuration other than superficial ulcerations is absolutely contraindicated.—*Dr. Gildersleeve, Cosmos,* 1875, p. 1355.

*Replacing a Bridge Facing.* "Select a facing of proper size and color. From very thin copper make a backing to fit the facing. This is merely to be used as a pattern, and we shall call it pattern number one. Next make a similar pattern of the space where the facing is to be replaced. Call this pattern number two. Place pattern number one over pattern number two and punch pinholes in number two, as indicated by number one. Place pattern number two (which now has pin holes in proper position), in space where facing has been lost, and using it as a guide drill pin holes through the backing left on the tooth, or bridge, as the case may be. Now place pattern number two on new facing and grind facing to conform with the pattern. Set facing in place and grind pins flush with the lingual surface of crown or bridge. Remove facing, thread the pins, and then with a large round bur countersink the holes on the lingual side. Set facing with cement and fill around pins with amalgam, and smile while you do it. The entire operation should be done in twenty minutes, and done well."—*Items of interest.*

*Malignant Tumors of the Jaws.* Turck believes with many others that cancer can be cured only by thorough operative removal in the early stages, and that cure is never certain unless the cause is removed before cancer begins. He believes that when cancer of the deep tissues has progressed to a point where a positive clinical diagnosis is possible, cure by any means whatever is improbable if not impossible.

In the vast majority of cases, the small growths, irritations, or tissue changes about the jaws from which cancer develops may be removed without danger under local anesthesia, with one hundred per cent. of cures. Cancer may be classed as a preventable disease; it always gives some warning of its approach; there is always first some small benign sore, ulcer, or growth; cure is always possible if the disease is arrested in its incipience. Procrastination has killed more patients than the surgeon's knife.

Turck states that differential clinical diagnosis between early malignant epulis and benign fibroma of the gum is usually impossible; the growth should be removed, subjected to immediate microscopic examination, and, if found malignant, radical resection should be done at once.—*Journ. Florida Med. Association,* per *Surgery, Gynecology, and Obstetrics.*

*Preserve the Pulp.* I am not one of those who hold the dental pulp in light esteem.. To say that it has no function and consequently no value in adult life is to betray a woeful ignorance of and lack of faith in the wisdom of an all-wise Creator. Nature does produce some freaks, but it is past believing that she would universally make the mistake of leaving the pulps in teeth past an age when there is nothing for them to do and when the teeth are as well or better off without them. The fact that pulps die or become diseased and have to be removed offers no adequate excuse for the indiscriminate destruction of normal pulps. Legs become gangrenous, kidneys diseased, and many other parts of our anatomy degenerate into pathological conditions that necessitate their removal; but the surgeon having a conscience does not remove these organs or parts except under the most serious provocation. The same principles should apply to the dental pulp. If we do not understand or appreciate its functions, we should be willing to lay the failure to lack of knowledge on our own part and not to lack of function on the part of the pulp. There is a mutual dependence of each of the tissues of the tooth upon each of the other tissues for its maintenance in the highest degree of health and service. And just as the eye can not say to the hand "I have no need of thee," so may not any tooth tissue say to any other tooth tissue "I have no need of thee."—Dr. Burgess in *Cosmos,* Dec. 1915, page 1337.

*Restoring Occlusal Surfaces With Amalgam.* "This method is applicable only to teeth in which the entire masticating surface is to be restored. I use the die forms intended for striking up occlusal cusps for gold shell crowns, and I strike up a large variety of cusps, using 36 guage copper. The cavity of the tooth having been prepared, I select a copper cusp of approximately correct size and form and place it upon the tooth to be filled, burnishing the margins of the copper cusps, close against the natural tooth, so that it may not be dislodged when the patient bites upon it. I then have the patient bring the jaws together and bite upon the copper cusp, which is soft enough to yeild under this stress, and thus is produced an accurate occlusion. The copper cusp is then inverted and thin tin foil burnished against the inner side, after which it is filled with amalgam. It is preferable to use one of the quick-setting types of amalgam, which is soft for a minute or two. The cavity is then quickly filled with same material and the copper cusp placed in position on the tooth so that the two amalgams come together and unite under pressure, which is best insured by having the patient close the jaws again. The mouth should be kept shut until the amalgam has begun to set, when the copper cusp can be lifted off and any excess removed. The result will give good reproduction of natural teeth and the occlusion will be accurate.''—*Items of Interest.*

*Dentist's Health.* There is no profession or vocational work which demands a higher type of man physically, mentally or morally than dentistry.

*Physically,* because the profession, due to its close confinement and little real physical exertion, requires a good strong animal type to be able to pull the required working years of an average life; and few will allow, due to the close physical position necessary to work, a bad breath, a bad breather, a diseased person, or one having a physical deformity, to long continue as their professional advisor: and right here one of the very best investments you can make is to learn to recreate—at golf, tennis, planting flowers, gardening, or be a chicken fancier. Most dentists try to work too many hours, mix too little, too much wear on the physical machine, hence become old at an early age. Learn to conserve your health.

*Mentally,* because keenness of judgment and quickness of action, a saver of time and the dentist's capital.

*Morally,* because no profession may lend more temptation to the unscrupulous person, and as professional men we must hold ourselves above reproach.

Some dentists don't have time to read, don't have time to even mail return post cards, don't have time to attend professional meetings, don't have time to recreate, hardly time to eat, don't have time to associate, no time to laugh, hardly time to go home at night.

Josh Billings says: "If a man kan't find time to laff, there is some mistake made in putting him together, and if he wont laff, he wants as much keepin' away from as a bear trap when it is sot."—Dr. McCullough, in *Summary.*

*The Relation of Dentistry to Medicine.* As one of the directors of the Research Institute of the National Association Dr. C. H. Mayo says, "I have become fairly familiar with the status of dentistry and its progress in America.. There is no other profession that is making more rapid scientific advancement.. The dentists are giving their work freely to the poor, and they have done much for preventive medicine by the inspection of school children whenever called upon.

"Minnesota, especially, is to be congratulated on the advanced position attained by the dental profession in both research and practical work, and this is very gratifying since it marks only a beginning in the great future of dentistry. The medical profession, as never before, has come to appreciate the work of the dentists. This is shown by the greater harmony, the association of work and the consultation between the two professions.

"There is great need for the establishment of free dental clinics throughout the state, and I see no reason why they should not be established in every city of 3,000 and upward. This would lead to better team work in the division of labor among physicians, to greater local harmony in the professions and would add to the appreciation of the dental profession by the public, besides being a common good.

"The dentists and the medical profession because of the newer ideas concerning disease, must of necessity become more closely associated in their work; the dentists must have more training in medicine and the medical man must have more and better training in the rudiments of dentistry, that consultation between the two professions may lead to increased good to the community. The time is not far distant when the public, by frequent or regular visits to dentists will seek prevention rather than cure in diseases of the teeth and jaws, with far reaching consequences in general and local diseases.''—*Summary*.

*Novocain Injection.* I am not going to enter into the technique of the different injections. There are books published by Drs. Guido Fischer and Kurt Hermann Thoma that explain every detail, but I want to recommend that every man who contemplates the use of conductive and infiltrative anesthesia should get one of these books, study it, then get someone who is thoroughly familiar with the technique to instruct him on patients, and if possible, do the different injections himself, under the guidance of his instructor.

There are two points I might mention in connection with the technique—one is enzymol. Enzymol is a sterile, purified solution of the gastric juice, extracted directly from the peptic glands of the fresh stomach, especially designed for local application. I use it to cleanse the mouth of thick, stringy saliva, and if a small injection is to be made, the part to be operated on can be swabbed off with a piece of cotton dipped in the enzymol solution. It cleanses the part thoroughly, so when you apply your iodin and aconite solution, you are sure to have a sterile field.

Another point I wish to emphasize is, not to rub the gum tissue directly over your injection, because the iodin and aconite solution dries the part and you can very easily break the tissue and form a field for infection, and sometimes a pretty sore mouth. Instead of rubbing the gum directly over the point of insertion, place your finger on the outside of the lip and you will get the same results without any possibility of breaking the gum tissue. The lip will move back and forth without causing any friction.

Without novocain in my equipment, I would feel at a very great disadvantage. Here let me confess ignorance regarding the care and use of a hypodermic syringe previous to Doctor Fischer's visit with me. I feel fortunate when looking back over the years I have used a syringe and have escaped inflicting serious injury on some patient. I am sure that many sloughing gums after extracting have been due to ignorance of proper asepsis. Conductive anesthesia should not be tried by the careless, dirty dentist—strict atention should be paid to all details of asepsis.—Dr. G. T. Gregg, in *Dental Summary.*

*Making Plaster Models.* The observation that a plaster cast is most easily separated from the impression either immediately after the plaster has set or else a considerable time afterward, is due to the behavior of plaster, which immediately after setting becomes very hard, but soon softens again owing to its giving off, after setting, a large quantity of water which has become superfluous when the setting reaction is complete. Plaster, in order to set, requires, according to the writer, two molecules of water. Since it is technically impracticable to add exactly two parts of water to plaster, as it would not be soft enough, an excess of water is used. After the plaster, in the process of setting, has bound the necessary water of crystallization, a considerable quantity of water is subsequently liberated, impairing the integrity of the cast. In setting, plaster undergoes an expansion of one per cent., this expansion being greatest during setting, the heat of the setting process causing additional increase in volume. Attempts to overcome this expansion by the addition of various substances, chiefly aluminum salts, have proved unsatisfactory; it seems best to avoid all admixtures.

The mixing is done best by pouring the water first in the plaster bowl and adding the plaster thereto until no water remains visible. Then an additional small quantity is added, which will at first remain dry, but will gradually absorb water. Little stirring is then necessary; the more vigorously and the longer plaster is stirred, the more it expands and the more slowly it sets. Impression plaster, of course, is mixed with more water, is stirred longer, and is hastened in its setting process by adding potassium sulfate or sodium chlorid to the water. As the impression and cast can not be made simultaneously, and the impression is considerably smaller in bulk than the cast, the impression will harden far more quickly than the cast, which retains a great deal of water, and this is prevented from rapid evaporation by the covering impression. It is essential, therefore, to saturate the impression with water for five minutes, before pouring the cast, regardless of the separating medium used. In regard to separating media, the writer absolutely rejects soap solution, as it interferes with the setting of the cast by diffusing into the soft casting plaster, and produces a mushy surface layer. He uses either castor oil, or preferably shellac sollution (one part shellac in ten parts alcohol), which, however, must be applied only after the impression has thoroughly dried, else it will not enter the pores of the plaster, but form a superficial layer of uneven thickness. The shellac should be about as thin as pure alcohol, and two or three coats should be applied. As soon as the shellac has been thoroughly absorbed, the impression is painted with a solution of one part of sandarac in ten parts of alcohol. This solution also must be very thin, and care is to be taken to spread the liquid evenly, as it no longer enters the pores of the plaster. In this manner separating is easy, and the cast will have a smooth surface. The sandarac coating, of course, must also be allowed to dry perfectly, else the sandarac will glue impression and cast together. The perfectly dry impression is now laid in water for five minutes, first for the above-mentioned reason, and secondly in order to avoid osmotic action between the dry impression and the liquid casting plaster. The excess of water of the freshly mixed casting plaster would penetrate the separating medium, loosen it, and produce a smeary surface on the cast. The quality of plaster used is, of course, of great importance, and even good plaster may have been spoiled in transit by absorbing moisture.—Carl Herber in *Cosmos,* Dec. 1915, page 1403.

*Local Anesthesia.* In Dr. Stern's recent article (Local Anesthesia in Operative Dentistry), published in the September, 1915, *Items of Interest,* there appear several conflicting statements that in my opinion need rectification.

First: The essayist, like so many other writers on this subject, persists in using and advocating the pumping motion of the syringe when performing the maxillary tuberosity and mandibular injections, and I suppose in the infraorbital as well. Now, this "moving the syringe backwards and forwards" as the essayist advises, this pumping motion was advocated several years ago, and its purpose was to prevent the needle-point from remaining in the artery, had it by ill-luck entered one. It becomes, however, immediately apparent to any one who stops to consider it that, should the needle luckily miss puncturing an artery (as it always does), upon its initial full insertion, the advocates of this technique would give it (the needle) fifteen to twenty more chances by their repeated jabs. Moreover, since the syringe is never held so steadily, nor the jaws so motionless as to prevent the needle from deviating upon each successive pump from its initial path, it follows that not only are the chances increased for the very thing they wish to avoid, but the chances for post-operative pain and trismus as a result of the laceration that the tissues are subjected to, are almost guaranteed.

I refrain from saying more than a word of the impossibility of this occurring accidentally, and of the difficulty

I have experienced in endeavoring to inject an artery in the living subject intentionally. The reason for this is, of course, due to the slippery resilient coat and to the comparative dullness of the ordinary needle. Of arterial anesthesia, Prof. Braun says, "it is of value in tubercular patients, in the aged with bronchitis and heart lesions, and other cases which are not suitable for general anesthesia."

The essayist on page 655 of the same issue writes as follows: "Infiltration anesthesia seems to involve a slight danger to the pulp, as may be observed in the following cases recorded, all injected for the purpose of preparing cavities where the dentine was highly sensitive. Most of these cavities were superficial.

| | | | |
|---|---|---|---|
| Infiltration .................... | 86 | 9 | 2 |
| Conductive .................... | 193 | None | None |

While the essayist employes the term "seems" in his text his table is without it, thus implying definiteness without qualification. The *"slight"* danger that seems to be involved, we read, is nothing short of hyperaemia or death of the pulp. He farther remarks, "unfortunately I have no data as to the condition of the pulps prior to the injection." Which is sufficient to clear and exonerate the infiltration method. Though I rarely employ this method it is not because it is in the least dangerous but rather because it is somewhat painful, the injections being made into hard, firm, taut gum tissue, whereas in the conductive regions the tissues are loose and cellular.

Novocain, characterized by its blandness whether injected perineurally, endoneurally or infiltrated, or when packed into an open wound will invariably permit the tissues to regain their normal state. In fact, novocain appears to hasten wound healing and even in operations for cleft-palate where undisturbed circulation is of the utmost importance, I have found it without fault. Furthermore, if a purposely infected solution be injected, say into the apical region of an incisor we would not only expect the death of the pulp but of the surrounding tissue. For, any contaminated solution passing through the lamella does not possess any *selective* action on the pulp, the pulp becoming affected only when the area concentric to it succumbs. The surrounding tissue remaining normal, one may rest assured that the pulp will not suffer.—S. L. Silverman. .

*Answers to State Board Questions.* There is no doubt in my mind that in the past the colleges have not been all they should have been. We all know that some of them have been very deficient in their courses of instruction. In other words, they have not given the student for his three years' work and expense all that the student was entitled to. The fact that we have badly educated dentists can not be blamed altogether upon lack of time for their proper instruction, but it must, in part at least, be blamed upon poor methods of instruction and inadequate equipment in some of our dental colleges.

In order to show just what kind of material is sometimes turned out by the colleges I want to read to you some of the answers given to questions asked in the examinations by the State Board here in Indiana last year.

To a question about normal respiration, one man answers: "The normal respiration should be forty in a minute, which I counted right in here." That poor chap was evidently very much excited.

"The Eustachian Tube is a small flexible tube about six inches long located back of windpipe, connects the œsophagus and stomach."

"The Eustachian Tube is a large duct emptying into the mouth; it squirts saliva into the mouth."

"Ptyalin is a substance found in blood."

"A physiological salt solution is one which may be formed by or from the juices and acids of the body."

In reply to the question about the Eustachian Tube, one man very honestly writes: "Forgotten even an idea of this one."

"Physiology is the science that treats of diseases and their causes.."

"The function of the Eustachian Tube is to take air into the lungs."

"The diaphragm is the bony framework consisting of ribs, sternum and vertebræ."

"Ptyalin is secreted by the pancreas and pancreatic juice."

"Chyme and chyle must be different names for some kind of enzymes, but I never heard of the names. Morrison never handed it out."

"The diaphragm is an oblong, round cavity in the trunk of the body containing heart, lungs, stomach, liver, kidneys, spleen, pancreas, large and small intestines. In fact, all vital organs."

"The diaphragm is a cavity containing thoracic and abdominal viscera."

"Ptyalin is a substance which is made by the fermentation of carbohydrates. Its function is to render the peptones digestible."

"The diaphragm is the bony part of the body."

"The diaphragm is maid (sic) up of the ribs, and gives shape to body. Its most important (sic) use in the protect (sic) hart (sic) lungs and other organs."

"The diaphragm is the framework of the body."

"A frenum is a little tit-like projection on the posterior part of the tongue, this is the principle one found in the mouth."

"The frenum linguæ are principle frena in the mouth, they are sort of openings or canals into the mouth."

"The function of the red blood cell is to promote infection and disease.."

"The upper first molar (permanent) has four cusps and two roots."

"Blood plasma becomes lymph when it comes in contact with the air."

"The nerve which supplies the right upper central incisor is the submaxillary division of the fifth."

"A frenum is connective tissue connecting the vertebræ."

"The Antrum of Highmore is a small fossa or opening just below the second lower molar and usually perphiated (sic) by the roots of the molar."

"The inferior maxillary is a very large bone containing sixteen prominent teeth if in normal occlude (sic). It forms the hard palate. It has no motion in mastication."

I could quote you answers like that almost indefinitely, but what's the use? You have had enough. There may have been one or two of these men who were not college men but men who had the privilege of re-examinations because they were applicants under the old law, but the others are all college men, some of them juniors who had passed their examinations on these subjects before the college examiners, the others, men who had been graduated and given diplomas.

Now I ask you: Were those men given full value for the time and money the college had taken from them? Undoubtedly not. The college instructors retort that they can not put brains into a head made empty by nature. And that also is true. They can not make students and they can not make dentists of men unfitted by nature to be students and dentists. But here comes the rub—the unfair rub: They pass these men, defective as they are, giving them certificates of competence when they are not competent. Is it honest?

It is just here that the good of a State Examining Board comes in. If all the colleges were ideally competent and ideally honest in their treatment of the profession and the student, then there would be no need of State Examining Boards, which are nothing more than police supervisors of the colleges.—H. C. Sexton, in *Dental Summary*.

